# Trunk Instability in the Pitch, Yaw, and Roll Planes during Clinical Balance Tests: Axis Differences and Correlations to vHIT Asymmetries Following Acute Unilateral Vestibular Loss

**DOI:** 10.3390/brainsci14070664

**Published:** 2024-06-29

**Authors:** John H. J. Allum, Claudia Candreia, Flurin Honegger

**Affiliations:** 1 Departments of ORL, University of Basel Hospital, 4031 Basel, Switzerland; flurin.honegger@usb.ch; 2 Cantonal Hospital, 6016 Luzern, Switzerland; claudia.candreia@luks.ch

**Keywords:** unilateral peripheral vestibular deficit, balance control, vestibulo-ocular reflex, video head impulse test, body-mounted gyroscopes, clinical stance and gait balance tasks

## Abstract

BACKGROUND: Clinical dynamic posturography concentrates on the pitch and roll but not on the yaw plane instability measures. This emphasis may not represent the axis instability observed in clinical stance and gait tasks for patients with balance deficits in comparison to healthy control (HC) subjects, nor the expected instability based on correlations with vestibulo-ocular reflex (VOR) deficits. To examine the axis stability changes with vestibular loss, we measured trunk sway in all three directions (pitch, roll, and yaw) during the stance and gait tasks of patients with acute unilateral vestibular neuritis (aUVN) and compared the results with those of HC. Concurrent changes in VORs were also examined and correlated with trunk balance deficits. METHODS: The results of 11 patients (mean age of 61 years) recorded within 6 days of aUVN onset were compared within those of 8 age-matched healthy controls (HCs). All subjects performed a two-legged stance task—standing with eyes closed on foam (s2ecf), a semi-gait task—walking eight tandem steps (tan8), and four gait tasks—walking 3 m with head rotating laterally, pitching, or eyes closed (w3hr, w3hp, w3ec), and walking over four barriers 24 cm high, spaced 1 m apart (barr). The tasks’ peak-to-peak yaw, pitch and roll angles, and angular velocities were measured with a gyroscope system (SwayStar^TM^) mounted at L1-3 and combined into three, axis-specific, balance control indexes (BCI), using angles (a) for the tandem gait and barriers task, and angular velocities (v) for all other tasks, as follows: axis BCI = (2 × 2ecf)v + 1.5 × (w3hr + w3hp + w3ec)v + (tan8 + 12 × barr)a. RESULTS: Yaw and pitch BCIs were significantly (*p* ≤ 0.004) greater (88 and 30%, respectively) than roll BCIs for aUVN patients. For HCs, only yaw but not pitch BCIs were greater (*p* = 0.002) than those of roll (72%). The order of BCI aUVN vs. HC differences was pitch, yaw, and roll at 55, 44, and 31%, respectively (*p* ≤ 0.002). This difference with respect to roll corresponded to the known greater yaw plane than roll plane asymmetry (40 vs. 22%) following aUVN based on VOR responses. However, the lower pitch plane asymmetry (3.5%) in VOR responses did not correspond with the pitch plane instability observed in the balance control tests. The increases in pitch plane instability in UVL subjects were, however, highly correlated with those of roll and yaw. CONCLUSIONS: These results indicate that greater yaw than pitch and roll trunk motion during clinical balance tasks is common for aUVN patients and HCs. However, aUVN leads to a larger increase in pitch than yaw plane instability and a smaller increase in roll plane instability. This difference with respect to roll corresponds to the known greater yaw plane than roll plane asymmetry (40 vs. 22%) following aUVN observed in VOR responses. However, the lower pitch plane asymmetry (3.5%) in VOR responses does not correspond with the enhanced movements in the pitch plane, observed in balance control tasks. Whether asymmetries in vestibular-evoked myogenic potentials (Vemps) are better correlated with the deficits in pitch plane balance control remains to be investigated. The current results provide a strong rationale for the clinical testing of directional specific balance responses, especially yaw and pitch, and the linking of balance results for yaw and roll to VOR asymmetries.

## 1. Introduction

If the motion of the eyes about each rotation axis (yaw, roll, and pitch) acts as a “speedometer” for subsequently driven postural vestibulospinal responses [1], then, following a peripheral vestibular deficit, the amplitudes of muscle responses and body motion comprising balance corrections should be correlated with the readings of the “speedometer”. That is, the vestibulo-ocular reflex (VOR) values measured with the video head impulse tests (vHIT) should be correlated with the motion amplitudes of the body’s center of mass during clinical stance and gait tasks or when the base of support is perturbed [2,3]. Our recent pilot study [4], examining motion of the lower trunk close to the center of mass, led to ambiguous results. The vHIT roll plane asymmetry was well correlated with the roll movements of the trunk, especially for gait tasks, such as walking with tandem steps. In contrast, no significant correlations were found for the vHIT pitch plane measures. The yaw plane motion was not examined in this pilot study [4].

The lack of correlation for the pitch plane could be related to mismatches between the signals provided by the utricular otoliths and the posterior semicircular canals on body angular tilt and velocity, respectively. For example, this mismatch is likely to occur following bilateral peripheral vestibular loss (BVL) for which the canal systems are usually 100% deficient. In contrast, 60% of these BVL patients have a remaining utricular dysfunction [5]. Thus, apart from rare cases where the canal system is intact but the otolith system is not [6], integrating the two systems’ responses together in the CNS [7,8] will yield a canal response in which the angular rotation about the posterior canal axis is underestimated, while the utricular responses signaling head tilt with respect to gravity is also underestimated, thus causing the translational head acceleration to be overestimated. This reasoning for the pitch plane cannot be applied in the case of aUVN where the superior nerve and, with it, the utricle responses are affected, leading to the underestimation of tilt responses, which would be in conflict with the correct estimates of head pitch angular velocities emanating from the posterior canal responses served by the unaffected inferior vestibular nerve.

In this follow-up study, we have attempted to measure the effect of an aUVL caused by UVN, at onset and 5 weeks later, on balance control in each of the three axes, yaw, pitch, and roll, during clinical stance and gait tasks. Correlations were explored between an axis-defined balance control index (axis BCI) and vHIT response asymmetries about the same axes. Within each axis, we compared values for aUVL patients and healthy controls (HC) in order to determine which axis had the greatest pathology on UVN onset. We included the yaw axis in order to perform the aforementioned comparison between the aUVL patients and the HCs and then repeated the pitch axis measurements to confirm the differences previously observed with respect to the roll axis [4].

## 2. Methods

### 2.1. Subjects

Patient data were gathered from two hospitals, University Hospital Basel and Cantonal Hospital Luzern, following a standardized clinical protocol. The data of this study were analyzed retrospectively. Ethical approval for the study was granted by the Ethics Committee of Northwest and Central Switzerland (EKNZ), with the project number 2014-026 for the University Hospital Basel and amendment 5, dated 25 January 2023, for the Cantonal Hospital Luzern. The principal investigator was JHJA.

The study focused on data from 11 patients, comprising 2 women and 9 men, with a mean age of 61.4 years (standard deviation (sd) 3.0 years, range 40 to 71 years), diagnosed with an acute unilateral peripheral vestibular deficit (aUPVD), presumed to be caused by unilateral vestibular neuritis (UVN). The diagnosis criteria included a pathological lateral canal paresis during caloric testing (mean of 94.4%, standard error of the mean (sem) of 3.0%, the upper 95% limit for healthy controls is 30%), as well as a pathological lateral vHIT gain (less than 0.74) corresponding to the affected canal paresis side, presence of spontaneous nystagmus beating towards the unaffected ear, nausea, and the persistence of symptoms over hours. In other words, the symptoms were required to fit the description of the diagnostic criteria for Acute Unilateral Vestibulopathy (AUVP), a synonym for acute unilateral vestibular neuritis (aUVN) as defined by the Committee for the Classification of Vestibular Disorders of the Barany Society [9]. Measurements were conducted at the onset of the aUPVD, approximately 3.2 days (sem 0.5 days) following the diagnosis of UVN. These measurements were repeated after 5 weeks, by which time the canal plane gain asymmetries had typically reached a common level of around 20%, due to the effect of central compensation on the VOR [10].

All patients received intravenous methylprednisolone treatment (125 mg Solumedrol per day, Pfizer, Switzerland) during their hospital stay. This was followed up four days after discharge, with the same medication taken orally. Written consent was obtained from all patients for the anonymous use of their data in publications.

Patients presenting with concurrent balance issues stemming from alternate origins such as peripheral lower-leg neuropathy, recent knee surgery, or signs of a central vestibular deficit were excluded from participation in this study. The responses of 8 healthy individuals, matched for age, were compared with the patient data.

### 2.2. Measurement Systems

A caloric examination was conducted to assess canal paresis and the level of unilateral weakness. A bithermal caloric test involving water temperatures of 44 °C and 30 °C was utilized. The comparison of average eye slow phase velocity (SPV) during the culmination phase of nystagmus was performed between irrigations of the left and right ears. Specifically, CP (Canal Paresis) was defined as ([R − L]/[R + L] × 100%), where R represents the algebraic difference in SPV levels between irrigation of the right ear with 44 °C and 30 °C water, and L denotes the corresponding difference for the left ear.

**Figure 1:**
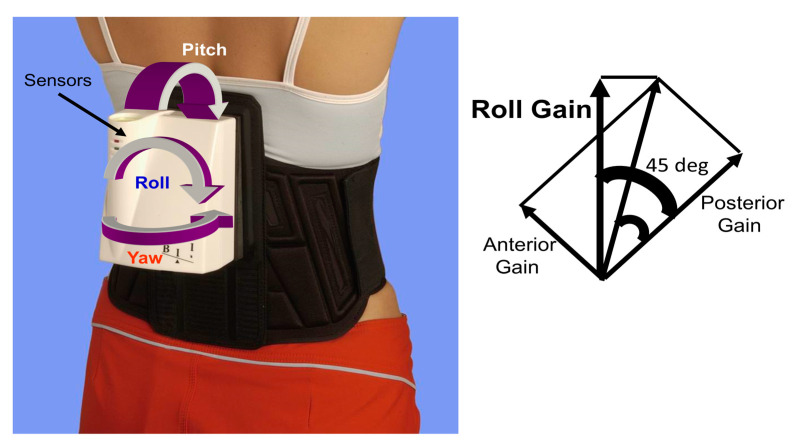
SwayStar equipment mounted on the back of a subject (**left**). On the (**right**) is a graphic of the trigonometry used to transform gains along anterior and posterior canal axes on the same side to along the body roll axis on the same side (see equation above).

To assess the VOR function in response to high angular accelerations exceeding 2000 degrees/s^2^ during head impulses, a video Head Impulse Test (vHIT) system was employed (ICS system from GN Otometrics, Natus Medical Inc., Taastrup, Denmark). The methodology followed the protocol outlined by MacDougall et al. [11], with the head angular velocities reaching 100 to 250 degs/s by 100 ms after onset of head velocity. A minimum of 15 head rotations with artifact-free responses were executed in each canal plane. All vHIT examinations were conducted by the same individual (FH), with the exception of those in Luzern (CC). During the head movements, patients were seated and fixated on a small target positioned 3m away. For the vertical canals, the head was initially rotated 45 degrees, in yaw, followed by upward or downward head rotations along the canal plane. Segments of the data containing covert saccades and artifacts were removed from the recordings before gain calculations using the vHIT manufacturer’s (GN Otometrics) software,(version 3). Gains were computed by measuring the quotient of the areas under the eye and head velocity–impulse responses. The calculation interval for this quotient commenced 100 ms prior to peak head velocity and was terminated when head velocity first crossed zero after this peak. These gains were then correlated with the peak-to-peak ranges of trunk pitch, yaw, and roll velocities measured using the SwayStar system. To perform this correlation, the vHIT canal gains were transformed into left and right roll gains according to the following equation (see trigonometry in Figure 1, right):

$$Roll\;gain=\frac{\sqrt{{ant}^{2}+{pos}^{2}}}{1.414}*\cos\left(45-{tan}^{-1}\left(\frac{ant}{pos}\right)\right)$$

where ant is the anterior canal gain, and pos is the posterior canal gain on the same side.

The anterior pitch gain was calculated in a similar manner by combining the gains from both the right and left anterior canals, and likewise for the posterior pitch gain. In each instance, the deficient vertical gain direction was presumed to align with that of the anterior pitch gain, as the gain of the anterior canal was lower than that of the posterior canal on the deficit side (see the Results section). The lateral canal gain was employed as is, without modification, to represent the yaw gain.

A SwayStar^TM^ system (Balance International Innovations GmbH, Switzerland) was employed to assess the participants’ balance control. Specifically, this gyroscopic system was affixed to the lower trunk at the lumbar 1–3 level using a modified elasticated motorcycle belt (see Figure 1 left). The system sampled lower-trunk angular velocity in the yaw, pitch, and roll planes at a rate of 1 KHz, enabling real-time calculation of angular displacements through trapezoid integration. The identical standard protocol involving 14 stance and gait tasks was used, as previously described, to evaluate balance control [2]. Participants carried out the balance tasks without shoes, performing stance tasks of standing on either one or two legs with their eyes either open or closed. Each stance task lasted for 20 s, or ended when the participants lost balance, or their non-stance foot touched the ground during the one-legged stance task. When performing the one-legged stance task, participants used their preferred leg. Except for the standing on one leg with eyes closed trial, all stance tasks were repeated on a foam support surface measuring 10 cm in thickness, 50 cm in width, 150 cm in length, with a density of 25 kg/m^3^. The 4 tests standing on 2 legs are identical to the mCTSIB test battery [12], except that the feet were set hip width apart and not together. Additionally, participants performed a semi-stance gait-like task, walking eight tandem steps, both on a normal, hard, floor and on the foam support surface, while observing their feet. Five gait tasks were conducted at the participants’ preferred speeds. These tasks included walking 3m with eyes closed, walking 3 m with eyes open while rotating the head left and right, or while pitching the head up and down. Another task involved walking up and down two steps, each 23 cm high, while the final task required walking over four low barriers, each 24 cm high and spaced 1m apart. Throughout all the trials, one or two spotters stood alongside participants, as needed, to prevent falls. The duration of each gait trial was recorded until completion or loss of balance. All balance tests were administered by the same individuals (FH or JHJA) following a standardized protocol. Balance control measures included tracking the 100% range of angular displacement and velocity in the roll, yaw, and pitch directions from each trial.

We used a modified Balance Control Index (axis BCI) to indicate the magnitude of balance instability, separately, in the yaw, pitch, and roll axes. The axis BCI is based on the BCI formula originally developed by Allum and Adkin, 2003 [2], as follows:

BCI = 2 × s2ecfpv + 1.5 × w3mphpv + 1.5 × w3mecpv + 20 × w3mecdur + 1.0 × w8tanra + 12 × stairsra

where pv is pitch peak-to-peak velocity range, ra is roll peak-to-peak angle range, and dur is duration. For the current study, the modified formula used was the following:

axis BCI = 2 × s2ecfqv + 1.5 × w3mphqv + 1.5 × w3mecqv + 1.5 × w3mrhqv + 1.0 × w8tanqa + 12 × barriersqa

where q is the axis chosen—pitch, yaw, or roll. In the modified formula, the velocity value for the head rotation task has been added to have all gait tasks included. The value for duration of the w3mec protocol was not included in axis BCI measure as this variable is not axis specific. Instead, the axis velocity for the w3mec task has been added as this is related to the task duration [2]. The stairs measurement angle value was replaced with that of the barriers task as the stairs task could not be completed by 2 acute UVL subjects as they were too unstable. The barriers task is similar to the stairs task, in that it is a complex gait task. However, in contrast to the stairs task, it was completed by all subjects. Similar to the stairs task, the w8tanf task could not be used for all aUVN subjects.

### 2.3. Data Analysis

**Figure 2:**
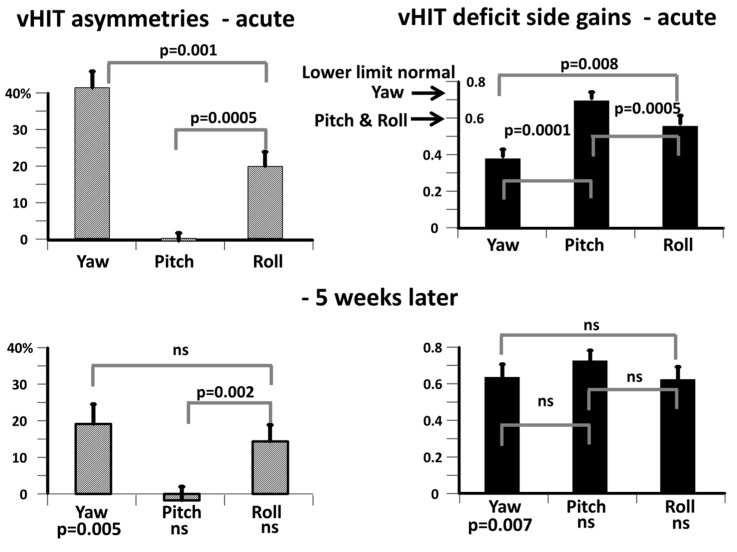
Population mean vHIT deficit side gains (on the **right**) and gain asymmetries (on the **left**) at onset of an acute UVN (upper sets of columns) and 5 weeks later (lower set of columns). As described in the Methods section, the gains have been transformed from the canal plane axes to the body plane axes used for measuring trunk sway. The lower normal limit of vHIT gain is marked by the arrows on the ordinate for acute deficit side gain. For yaw, this is the same as the lower limit of normal for the lateral canal gain, 0.74 [13,14]. As the lower limit is equal for anterior and posterior canals, 0.6 [14], this leads to an equal limit for roll and pitch. The height of each column in the figure represents the mean population gain. The vertical bar on the column represents the standard error of the mean (sem). Note the unchanged pitch gain and asymmetry indicating that the posterior nerve is, on average, not affected by the aUVN. Probability (p) of the significance between axis values is indicated by the values on the horizontal bar linking the compared axis values. ns stands for not significant. *p* values listed below the 5 weeks after onset columns indicate the significance of differences of axis values between onset and 5 weeks later.

**Figure 3:**
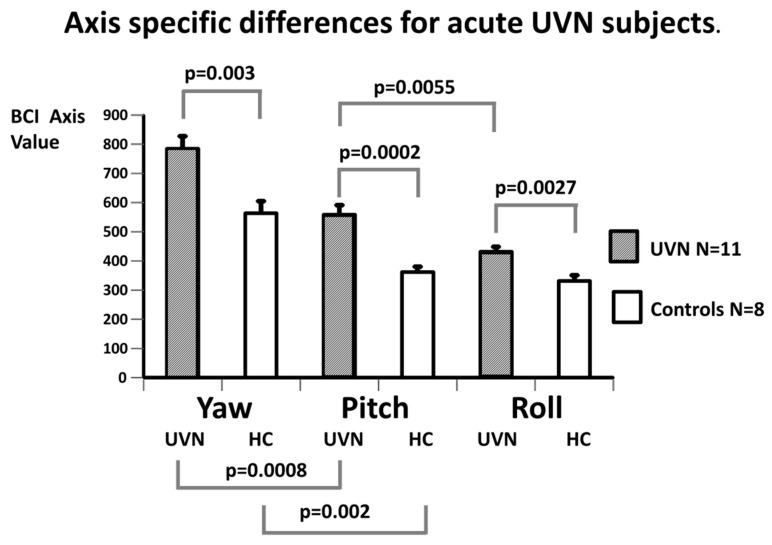
Axis-specific population differences in balance measures (BCI values) for acute UVN subjects. The height of each column represents the mean BCI values and the bar on each column the sem. Significant differences between onset aUVN and controls (*p* ≤ 0.003) for each axis are indicated by *p*-values from *t*-tests. Likewise, the axis differences within the aUVN and healthy control populations are indicated (*p* ≤ 0.0055). There was no significant difference between pitch and roll BCI values of healthy controls (not marked with a *p* value).

The 3 canal vHIT gains, the vHIT gain asymmetries, and the 3 yaw, pitch, and roll axis BCI values were compared pairwise between the set of 3 values using a paired *t*-test. The significance values quoted for the three comparisons in the Results section also holds true if the levels are Bonferroni corrected for multiple comparison because when a significance was present, its probability value, p, was always less than 0.05/3 = 0.0166, specifically less than 0.008 (see Figure 2 and Figure 3). Linear regression techniques were used to explore the correlations between vHIT gains and axis BCI values for the same yaw, pitch, and roll axes of trunk sway. The resulting Pearson’s correlation coefficients were considered significant if *p* < 0.05.

## 3. Results

### 3.1. vHIT Deficit-Side Gains and Asymmetries at Onset of aUVN and 5 Weeks Later

We examined changes in the video head impulse test (vHIT)-measured VOR gains and asymmetries in the three body planes, yaw, pitch, and roll, in order to determine whether the VOR gain changes with respect to normal, between the acute onset of UVN and 5 weeks later, were similar for the 11 aUVN subjects of the current study compared to the 20 subjects of our previous publication [4]. As shown in Figure 2 (right) and Table 1, the yaw axis vHIT gain was most reduced by aUVN, then the roll axis, followed by the pitch axis. Five weeks later, there was no significant difference between the gains for each axis (Figure 2, lower right and Table 1, columns 5, 6 and 7. Over the 5 weeks, the greatest change occurred for the yaw axis (*p* = 0.007). These gain changes are, however, highly dependent on the number of deficit-side posterior and anterior canal gains included in the averaging process [4]. In the current study, only 2 of 11 subjects had deficit-side posterior canal gains less than the lower limit of normality (0.6, see [13,14]), and 8 of 11 had deficit side anterior canal gains less than 0.6, whereas all 11 deficit-side lateral canal gains were less than normal (0.74, see [13,14]). Thus, the yaw asymmetry, which corresponds to the lateral canal asymmetry, had the highest asymmetry in the acute phase, but this was reduced to ca 20% at 5 weeks (see Figure 2, left). Inclusion of all the pitch gain values resulted in low vHIT asymmetry values, whereas the vHIT roll asymmetry values were intermediate between those of pitch and yaw, specifically, at 5 weeks ca. 20%, and not different from those of yaw [4]. Thus, as explained in detail below, viewing these different changes in vHIT gains and asymmetries with measurement axes and assuming that the same vestibular sensory signals that underlie pathological asymmetric vHIT gain values also underlie pathological balance corrections, it was counter-intuitive to us that we saw a greater increase in kinematics in the pitch plane, when compared to HCs, than in the yaw and roll planes.

### 3.2. Trunk Balance Control at Acute Onset of UVN and 5 Weeks Later

**Figure 4:**
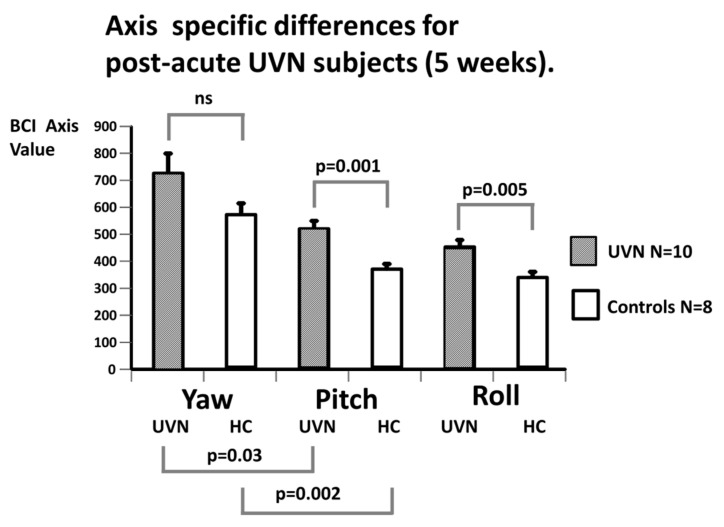
Axis-specific population differences in balance measures (axis BCI values) for acute UVN subjects 5 weeks after onset of symptoms. There was no significant difference between pitch and roll BCI values of aUVN subjects at 5 weeks. The layout of the figure is identical to that of Figure 3.

Figure 3 and Table 2 and Table 3 illustrate the mean value of the axis balance control index (axis BCI as defined in the Methods section) for each axis (yaw, pitch and roll). The means were calculated for acute UVN subjects and compared with the values of the HC. For both the UVN subjects and the HC, the yaw axis provided the largest, in absolute terms, trunk sway axis BCI values, while the pitch BCIs provided lower values, and the lowest values were for the roll axis. For each axis, the acute UVN values were significantly greater than those of the HCs and, interestingly, the most statistically significant population differences (*p* = 0.0002) occurred for the pitch axis. That is, the pitch axis trunk sway was most unstable with respect to HC. Figure 3 and Figure 4 and Table 1 and Table 2 compare the population means.

In Figure 4 and Table 3, the axis BCI values of the study populations, aUVN, at 5 weeks after onset of the UVN, and the HC subjects, are displayed. The order of kinematic values around an axis was the same at 5 weeks as at UVN onset, where yaw provided the largest trunk sway axis and roll the least sway. Again, the most significant difference between the UVN subjects and HCs occurred for the pitch axis (see Figure 4, and Table 2 and Table 3). A population difference with respect to the HCs was, however, still present for the roll axis (*p* = 0.005, see Figure 4). In contrast, the axis BCI values for the yaw axis were no longer significantly different from those of the HCs (Figure 4). The pitch and roll BCI values for the UVN population were also not significantly different from each other at 5 weeks. In addition, the UVN population values for each axis were numerically, but not significantly, less at 5 weeks compared to the values at aUVN onset (see Table 3).

Having established that our BCI values were different between axes, with most significant differences between UVN patients and the HCs occurring for the pitch plane, we next examined for which balance tests the population differences were occurring. The result of this analysis is presented in Table 2 and Table 3 and shows that across all gait task measures present in the axis BCI, those for pitch had the highest significant difference with respect to the HCs. The exception was for the gait task of walking over low barriers, which had a low significance value (*p* = 0.048). For the stance task, s2ecf, the significance was marginally higher for roll than pitch (0.0026 vs. 0.004).

### 3.3. Correlations between VOR and Trunk Sway Measures

**Figure 5:**
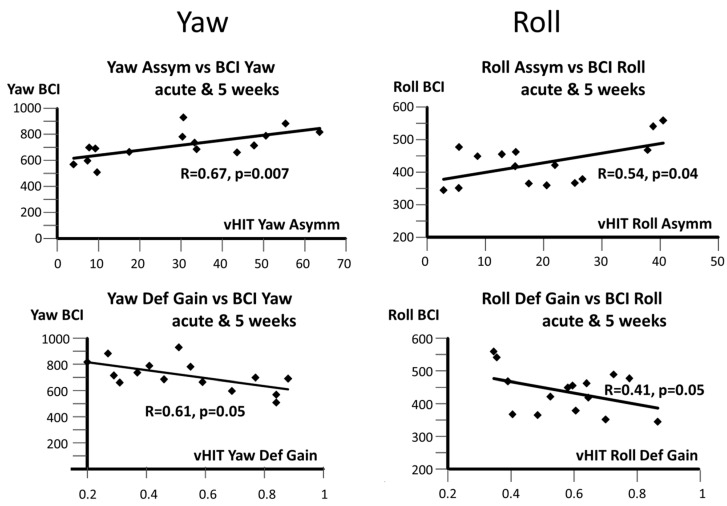
Correlations between axis-specific BCI values and vHIT roll and yaw asymmetries (upper plots). The lower plots show the correlations between BCI values and vHIT roll and yaw deficit-side gains. The data points for the regressions are shown as diamonds. Data were from patients measured within 5 days of acute onset of UVN symptoms and 5 weeks later. Note that the vHIT asymmetry regressions plots have larger and more significant regressions than those of the vHIT deficit-side gains. Regression plots for the pitch axis are not shown as these were not significant. The slope coefficient and its 95% confidence intervals for the regressions are as follows: (0.038, 0.12, 0.19) for the upper left plot, and (0.006, 0.1, 0.19) for the upper right plot, in order of magnitude. For the lower left and lower right plots, the corresponding values are (−0.002, −0.0012, −0.00028) and (−403.0, −173.0, −58.0), respectively.

Our working assumption was that the summary of the trunk sway balance measures combined from different tasks (axis BCI) would be correlated with the VOR measures in the same plane. Specifically, given the greater significance of balance measures in the pitch stance and gait tasks with respect to the HCs (Table 1 and Table 3), we expected that the correlations with the VOR deficit-side gain or gain asymmetry would be highest for the pitch plane as well. Remarkably, the correlations for the pitch plane (R = 0.19) were not significant. However, regressions for the yaw and roll planes were significant between the vHIT VOR asymmetry and axis BCI values, with R = 0.67 (*p* = 0.007) and R = 0.54 (*p* = 0.04), respectively, (see Figure 5). The correlations between the vHIT deficit gain and axis BCI values for the yaw and roll planes were less significant, as can be seen from the lower plots in Figure 4. In particular, the roll plane was of borderline significance (see also 95% confidence intervals in the legend of Figure 5).

**Figure 6:**
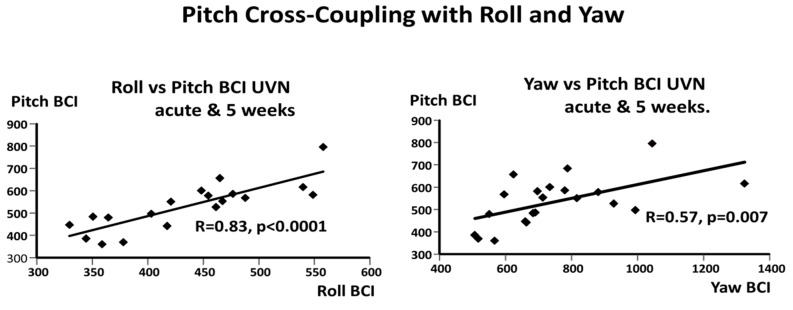
Correlations between roll and pitch BCI values (on the **left**) and yaw and pitch BCI values (on the **right**). The slope coefficient and its 95% confidence intervals for the regressions are (0.82, 1.26, and 1.7) on the left, and (0.1, 0.31, 0.52) on the right, in order of magnitude. Data were from patients measured within 5 days of acute onset of UVN symptoms and 5 weeks later.

Based on the correlation results described above, we considered the possibility that a cross-axis coupling effect could be occurring during the balance corrections. For example, when the knee is flexed during balance corrections, both trunk roll and pitch occurs [15]. Similarly, upper trunk rotation in roll induces an additional trunk pitch movement [16]. As illustrated in Figure 6, the resulting cross-coupled correlations were highly significant regressions, with the most significant occurring between the roll and pitch BCIs (R = 0.83, *p* < 0.0001).

## 4. Discussion

### 4.1. Differences between Balance Deficit Measures for Each Body Axis Compared to vHIT VOR Gain Asymmetries

The current results indicate that changes recorded in the balance measures for the pitch plane following aUVN are not correlated with changes in VOR pitch plane response asymmetries. In contrast, vHIT gain asymmetries and, to a lesser extent, deficit side gains (see Figure 5) for the roll and yaw planes are correlated with the roll and yaw balance measures, respectively. This result confirms our findings for the pitch and roll planes described in our previous publication [4]. Furthermore, the new results for the yaw plane indicate that the correlations between the VOR asymmetries and balance control measures are very similar for the roll and yaw axes. In a sense, it is not surprising that there is a lack of correlation for the pitch plane, given that VOR pitch plane deficit gain and therefore asymmetries are hardly affected by UVN (see Figure 2 and Table 1, and Allum and Honegger [4]) as the changes in VOR asymmetries with onset of aUVN are mostly due to changes in neural responses of the superior vestibular nerve serving the lateral and anterior canals. Thus, one of the questions arising with aUVN is whether both branches, posterior and inferior, of the vestibular nerve are equally affected by the disease. Based on a pathologic gain threshold of less than 0.6 (Taylor et al. [17]), only 2 of our 11 patients had pathological posterior deficit canal vHIT gains, whereas the corresponding numbers for the anterior (lower limit HC gain 0.6) and lateral (lower limit 0.74) canal gains were 8 and 11 of 11, respectively, suggesting a differing effect of aUVN across canal neural circuits occurs. A similar spread of the effect of acute UVN on the canal responses was noted by Taylor et al. [17]. In particular, this difference between posterior and lateral canal gains following UVN has been explained in two ways. Firstly, it has been argued that the anatomical characteristics of the bone structure surrounding the superior nerve, a longer bony tunnel and more bony spicules, predisposes the superior vestibular nerve to being more likely to be entrapped and susceptible to ischemia [18]. Secondly, there is evidence from low acceleration (20 deg/s^2^) whole-body rotations that VOR gain recovery is faster in the pitch (posterior) compared to the yaw (lateral canal) plane [19]. However, there are contrasting viewpoints based on the recovery for the vHIT tests. Büki et al. [20] argued that there was slower recovery and Lee et al. [21] faster recovery for posterior compared to the lateral and anterior deficit canal gains. Despite these explanations, the essential question still remains as to why the mean deficit-side VOR canal gain after the onset of UVN is, on average, higher, that is, less affected for the posterior canals. Yet, in contrast, the effect of aUVN on the trunk pitch sway during the balance tests is stronger than for the roll and yaw axes, that is, more and not less trunk movement is observed (Figure 3 and Table 1).

As far as we are aware, apart from our 2020 publication [4], there are no studies which have looked at correlations between VOR and VSR measures. Most work to date have examined the simultaneous effects on vestibularly evoked myogenic potentials (Vemps) in different body locations (eye, neck, and lower leg) elicited by tone bursts presented at the ears [22]. Alternatively, direct stimulation of the vestibular nerve has been employed [23]. These studies did not examine correlations between Vemp measures at different muscles and VOR response measures. Rather, the focus was on determining if a Vemp or VOR could be elicited and how variations in the stimulus parameters altered the response size.

The greater pitch than roll trunk sway we observed (see Figure 3) could also be due to changed neural responses from the otolith organs, which we did not examine, rather than from the influence of UVN on canal neural systems. These otolith receptor systems, utriculus and sacculus, are served by the superior and inferior nerves, and they respond to the roll and pitch tilt, respectively [24,25]. Curthoys and colleagues [26,27] showed that a 500 Hz tone-burst auditory stimulation evokes potentials (cVemps) in the ipsilateral sternocleidomastoid muscle via the inferior vestibular nerve. The same stimulation evokes potentials (ocular or oVemps) in the contralateral inferior oblique eye muscle via the superior vestibular nerve. The same research group showed that unilateral vestibular loss results in reduced or absent contralateral oVemp n10 response amplitudes and a reduced or absent ipsilateral cVemp p13-n23 amplitudes [26,27]. Combining these potential changes into asymmetry ratios [28] has enabled research groups to identify differences between asymmetries for different patient groups [29] and between patient groups and healthy controls [30]. Furthermore, examining patients with abnormal unilateral saccular or inferior vestibular nerve function (i.e., abnormal cVEMPs) has demonstrated significantly impaired postural control in the pitch plane as measured with pitch plane support surface rotations when compared to normal subjects [31]. However, this group with isolated decreased cVemps demonstrated significantly better postural stability (less trunk sway) when compared to the group with abnormal caloric responses, that is, with a lateral canal deficit [31]. Similarly patients with isolated unilateral oVEMP abnormalities due to utricular and superior nerve deficits demonstrate an increased prevalence of postural instability and swaying sensation [32]. It should, however, be recalled that these studies of Jacobson and colleagues [31,32] involved large scale studies of centrally compensated acute UVN patients and are less likely to reveal a postural stability characterizing the vestibular impairments associated with isolated unilateral utricular or saccular dysfunction following acute UVN. The step that needs to be taken in future studies is to examine the correlations between Vemp asymmetries and balance measures (for example, the axis BCI used in this research) in order to determine, as we have attempted with VOR gain asymmetries, if especially the pitch and roll plane balance deficits are correlated with cVemp and oVemp asymmetries, respectively. A correlation of balance measures with Vemp asymmetries but not VOR gain asymmetries may provide the basis for a defective central processing of angular and linear motion signals, yielding an incorrect calculation of required body pitch angle and pitch angular velocity for stability [33].

### 4.2. Biomechanical Cross-Coupling Effects between Body Axes

We noted a strong cross-coupling effect between the pitch and roll and between the pitch and yaw motion amplitudes expressed in the axis BCI values (see Figure 6), suggesting that pitch instability could be the result of Coriolis forces acting on the pitch plane via rotations in the yaw and roll planes [34]. That is, the natural balance stabilizing movements in yaw could destabilize those in pitch [35], particularly if the central processing of the pitch motion is defective. The destabilizing effects of coupling are difficult to avoid as flexing the knee to correct roll motion also causes trunk pitch motion [15], likewise for the lateral motion of the trunk [16]. Future studies therefore need to take both the central processing and biomechanical effects into account when seeking explanations for absent correlations between pitch plane neural signals of the VOR and balance instability.

### 4.3. Source of Enhanced Trunk Sway Kinematics in Pitch

One of the questions we have attempted to answer in this study is, which is the correlated VOR variable to pitch instability following the onset of aUVN? The lack of a correlation to the pitch plane VOR responses is in contrast with the evidence described above, that the inferior vestibular nerve is less affected by VN. Therefore, it would be expected that posterior canal and sacculus afferent fibers are also less affected. Therefore, the most parsimonious explanation for the lack of correlation is that deficient utricular responses to pitch plane tilt are the cause of pitch plane instability. In the absence of accurate information on the pitch tilt normally provided by the utricules [36], central vestibular processing is no longer able to process pitch-directed canal and otolith responses to distinguish between the gravitational and inertial components of pitch movements [36]. Presumably, this malfunction leads to pitch plane instability. Thus, in general, when analyzing relationships between eye movement, vHIT asymmetries, and trunk pitch velocity during balance control tasks, it may be necessary to correct the data for pathological pitch angle information provided by the otoliths.

A simplification we have made in this study concerns the assumed canal orientations we have used when transforming vHIT gains from a canal-based to a head-based coordinate system. While the assumption that vertical canal planes on the same side are at 90 degrees to one another can be accepted, there is a 21 degree difference in orientation between the vertical canal planes (left anterior versus right posterior) on opposite sides [37]. Furthermore, the lateral canal planes are not oriented horizontally, and the actual orientation could lead to a change in the VOR gain in the yaw plane and thus lead to a contribution to roll and pitch HIT gains. The tests, however, indicate no change in vHIT gain when the lateral semicircular canals are orientated in the horizontal plane compared with the plane with normal head orientation [38].

### 4.4. Gender and Age Considerations for Future Studies

Our suggestion for future studies is to investigate the relationship between Vemp amplitudes and pitch balance control in order to determine possible correlates between the two. The current study has limitations for which the question arises as to whether the effect of gender and age on Vemps needs to be taken into account when examining Vemp amplitudes. The gender ratio of male to female (4.5) is higher in our group than the expected ratio, 1.3, based on a recent study with 198 UVN patients [39]. Currently, we have no explanation for this difference in gender ratios. However, this difference may not be crucial. Reports to date have indicated significant gender effects on the amplitudes of o-Vemps [40] but not on the asymmetry ratio of o-Vemps and c-Vemps [40,41]. Thus, our recommendation to correlate Vemp amplitude asymmetries with pitch axis balance control is probably not affected by gender effects, provided the asymmetry ratios are used. Other demographic aspects, such as age, do need to be taken into account, but were not a limitation for the current study. All patients were between the ages of 40 and 71 in the current study, and 90% were in the study of Allum and Honegger [4]. A similar age range was found for UVN patients by Mandala et al. [39]. Given this age range and the effects of age on Vemp amplitudes [42,43], specialized frequency techniques may need to be used to enhance the detection of cervical Vemps [44].

## 5. Conclusions

The primary aim of this research was to establish, in acute UVN patients, whether a relationship exists between the changed yaw, roll, and pitch vHIT gain asymmetries during recovery for UVN, and the changed yaw, roll and pitch trunk velocities and angles, respectively, during balance tasks. We could confirm such a relationship for the trunk yaw and roll angles and the angular velocities measured during the stance and gait tasks, as well as when these measures were combined into our balance control index (axis BCI) measure. Interestingly, we could not confirm this relationship for pitch, despite the pitch axis measures comprising the axis BCI values having significantly greater values than those of the healthy controls for stance and gait tasks (Table 1). One reason for this lack of correlation may be due to the small effect of acute UVN on the pitch vHIT gain asymmetries but a larger effect on the utricle responses [32]. In contrast, the yaw and roll balance instability can be predicted from these vHIT measures. Interestingly, measures of otolith function in the form of c-Vemps appear to track improvement in the UVN symptoms [45]. The question is whether c-Vemps track improvements in pitch-directed balance control as well. Future work should also investigate how this information could be employed to aid therapy for different types of UVL patients. This latter suggestion begs an answer to the question concerning the source of the sensory deficit for unstable pitch balance control following the onset of acute UVN and suggests that VOR pitch plane responses are not acting as speedometers for pitch plane balance corrections, even if those of the yaw and roll planes are apparently in their respective planes.

## Figures and Tables

**Table 1:** Axis-specific mean vHIT asymmetries and deficit side gains at aUVN onset and 5 weeks later are listed in rows 3 and 4 of the table in bold text. The standard error of the means (sem) is listed after each mean value. Significant differences between acute UVN means across axes and values at 5 weeks across axes are illustrated in Figure 2 as are significant differences between means at aUVN onset and 5 weeks later.

	vHIT Mean Asymmetry (sem)			vHIT Deficit Side Mean Gain (sem)		
	Yaw	Pitch	Roll	Yaw	Pitch	Roll
Acute	**41.55** (4.04)	**−0.73** (2.22)	**19.98** (4.01)	**0.38** (0.04)	**0.69** (0.03)	**0.56** (0.04)
+5 weeks	**19.14** (5.45)	**−1.79** (3.73)	**14.35** (4.5)	**0.64** (0.07)	**0.73** (0.06)	**0.63** (0.07)

**Table 2:** Axis-specific significant differences between acute UVN and healthy control subjects for clinical stance and gait tasks.

Task and Measure	Yaw	Pitch	Roll
s2ecf**v**	**0.012**	**0.004**	**0.0026** ●
wtan8**a**	**0.0012**	**<0.0001** ●	**<0.0001**
barriers**a**	**0.048** ●	0.04 1-sided	0.03 1-sided
w3mhr**v**	**0.021**	**0.0035** ●	**0.03**
w3mhp**v**	ns	**0.0002** ●	**0.008**
w3mec**v**	ns	**0.036** ●	ns
axis BCI	**0.003**	**0.0002** ●	**0.0027**

Table 2 lists the 2-sided *t*-test probabilities of significant differences between aUVN and healthy control population means for each protocol used for computing the axis BCI as described in the methods section. Significant 1-sided *t*-test probabilities were entered in the table when 2-sided tests were not significant. ns stands for 1-sided *t*-test not significant. The symbol ● was used to mark the axis for the listed test protocols and axis BCIs with the highest statistical significance. The following abbreviations have been used for the test type: s2—standing on 2 legs; ec—eyes closed; f—foam; w—walk; tan8—8 tandem steps; m—metres; hr—head rotating; hp—head pitching, and for the measures **a**—angle; **v** angular velocity.

**Table 3:** Axis-specific population means (bold text) and standard errors of mean (sem) of trunk sway for acute UVN, aUVN patients, 5 weeks later, and healthy control subjects for selected clinical stance and gait tasks comprising the axis BCI. Significant differences between the means are presented in Table 2.

	Axis Balance Control Index (sem)			Standing on 2 Legs on Foam Vel (sem)		
Population	Yaw	Pitch	Roll	Yaw	Pitch	Roll
AcuteUVN	**783.4** (43.1)	**557.8** (33.2)	**431.2** (21.6)	**24.8** (5.4)	**23.3** (4.4)	**12.7** (2.1)
+5 weeks	**714.9** (73.1)	**510.4** (29.1)	**442.8** (25.7)	**17.2** (8.5)	**13.9** (4.2)	**7.8** (2.03)
Controls	**562.5** (42.1)	**361.5** (52.3)	**330.8** (20.1)	**7.7** (1.0)	**5.7** (0.6)	**3.9** (0.5)
	**Walking 8 tandem steps ang (sem)**			**Walk 3m with head pitching vel (sem)**		
**Population**	**Yaw**	**Pitch**	**Roll**	**Yaw**	**Pitch**	**Roll**
AcuteUVN	**25.0** (2.7)	**14.8** (1.0)	**15.7** (1.1)	**83.0** (7.5)	**76.7** (2.6)	**51.9** (3.0)
+5 weeks	**12.2** (1.5)	**10.7** (0.6)	**10.1** (1.2)	**88.9** (8.2)	**65.6** (5.2)	**57.9** (4.0)
Controls	**11.7** (1.7)	**6.2** (0.5)	**7.7** (0.8)	**68.8** (5.7)	**50.8** (4.2)	**39.5** (2.5)

## Data Availability

The analysis data will be made available to those requesting it.
